# Lithotrity Performed on the Same Patient Forty-eight Times

**Published:** 1854-04

**Authors:** 


Lithotrity Performed on the same Patient forty-eight Times. —Mr. Coulson exhibited the bladder taken from a man aged eighty-three, on whom lithotrity had been performed forty-eight times during twenty years. Whether one or more fragments of the original calculus may have been left in the bladder, and became neuclei of secondary formations, or whether the bladder was at first completely freed, and the relapses depended on the same constitutional disposition which gave rise originally to the deposit of calculous matter from the urine, Mr. Coulson was unable to say. He did not see the case until the middle of last year. It cannot be denied that relapse occurs more frequently after lithotrity than lithotomy. The Norwich tables of Mr. Crosse show twelve cases of relapse after 704 operations of lithotomy, or 1 in 58. From records of operations performed at the Hospital of La Charité, in Paris, between the years 1806 and 1831, it appears that 6 cases of relapse presented themselves after 70 cases of lithotomy, or about 1 in 11. M. Civiale states that the proportion indicated by returns which he received from Bavaria, is 5 in 162 operations (1 in 32); from Bohemia, 1 in 46 operations; from Dalmatia, 1 in 43 operations. At the Luneville Hospital, founded by Stanislaus, King of Poland, for the treatment of calculous disorders, the register shows 13 cases of relapse after 1492 operations of lithotomy, or 1 in 116 cases. The most correct registers are probably those kept at the Norwich and Luneville Hospitals, and from them it appears that relapse occurred after lithotomy once in 58 cases at Norwich, and once in 116 cases at Luneville. For lithotrity there are no other data than those furnished by M. Civiale from his own practice. After 548 operations, relapse followed in fifty-five cases, giving a proportion of nearly 1 in every 10 cases. After lithotrity, every tenth patient may suffer relapse; after lithotomy, the proportion seems reduced to 1 in 60. Mr. Coulson did not think sufficient attention was paid to the condition of the urine after lithotrity, as an indication of the existence of fragments in the bladder. It is not enough that the painful symptoms produced by the stone shall have subsided, and no portion can be detected by the sound or lithotrite. If the urine when first passed is at all turbid, and especially if it contains any exudation corpuscles, first pointed out by Dr. Golding Bird, there can be little doubt that some fragment remains behind. This distinguished physician laid great stress on these bodies, as indicating the presence of stone, and Mr. Coulson knew two cases lately, in which, in consequence of the existence of these corpuscles, persevering efforts were successfully made in search of stone, although on previous examinations none could be detected. Mr. Coulson then gave the following description of these bodies, for which he expressed his obligation to Dr. Golding Bird: "These large organic globules can hardly be distinguished under the microscope from pus globules. They exhibit several nuclei on the addition of acetic acid. To those unacquainted with microscopic observations, the following characters of the urine will afford excellent and safe guides. The urine is pale; looks as if a little gum had been deposited in it, an appearance quite characteristic on agitation. Specific gravity lower than normal; does not coagulate by heat, but on addition of liquor potassæ, in a quantity equal to one-third the bulk of urine in a test tube, the whole becomes quite transparent, and more or less gelatinous, sometimes so sizy as to be pourod out with great difficulty. The differential diagnosis from purulent urine is the absence of albumen, and from mucous urine its gelatinization with liquor potassæ." To revert to the case now before the Society, the patient, aged eighty-three, on whom lithotrity had been performed, in the last twenty years, forty times, was sent by Mr. Wheeler, of Chelmsford, in May last, to Mr. Coulson, who at eight sittings crushed, without the least difficulty, a large lithic acid calculus, the detritus which came away weighing an ounce. The operations were not attended with any unfavorable symptoms, and on the 9th of June he returned home. Towards the end of the year, symptoms of vesical irritation returned, and Mr. Wheeler again sent the patient to Mr. Coulson, who admitted him on the 26th of November under his care at St. Mary's Hospital. He then complained of frequent desire to pass urine, and pain at the neck of the bladder and in the perinæum after voiding it. The urine contained some mucus, but no pus, blood, or albumen. There was no doubt that either a portion of the original calculus remained, or a new calculus had descended. Four ounces of tepid water having been injected, the lithotrite was introduced, and passed over a calculus on the right side of the bladder, but by no manipulation or change of posture of the patient could the stone be seized between the blades of the lithotrite, or the instrument be passed behind the calculus. The trial was made on two different occasions under chloroform by Mr. Coulson and others. As there were no urgent symptoms, it was determined to wait, but the man became impatient at the delay, and returned home on the 10th of December. He got cold, and died on the 30th of December.—*Lancet.*

